# Effect of tipiracil hydrochloride, thymidine phosphorylase inhibitor, on the ischemia/reperfusion injury of brain tissue in rats

**DOI:** 10.1038/s41598-026-51551-6

**Published:** 2026-05-20

**Authors:** Małgorzata Trocha, Tomasz Piasecki, Paulina Nowotarska, Tomasz Sozański, Anna Merwid-Ląd, Beata Nowak, Marcin Nowak, Rafał Ciaputa, Grzegorz Mazur, Adam Szeląg, Damian Gajecki, Adrian Doroszko

**Affiliations:** 1 Clinical Department of Diabetology, Hypertension and Internal Disease, Wroclaw Medical University, Borowska 213 Str, 50-556 Wroclaw, Poland; 2 Department of Epizootiology With Exotic Animal and Bird Clinic, Wroclaw University of Environmental and Life Sciences, Grunwaldzki Sq. 45, 50-366 Wroclaw, Poland; 3 Department of Biostructure and Animal Physiology, Wroclaw University of Environmental and Life Sciences, Norwida 31 Str, 50-375 Wroclaw, Poland; 4 Department of Preclinical Sciences, Pharmacology and Medical Diagnostics, Wroclaw University of Science and Technology, Hoene-Wrońskiego 13 C Str, 58-376 Wroclaw, Poland; 5 Department of Pharmacology, Faculty of Medicine, Wroclaw Medical University, Mikulicza-Radeckiego 2 Str, 50‐345 Wroclaw, Poland; 6 Department of Pathology, University of Environmental and Life Sciences, Norwida 31 Str, 50-375 Wroclaw, Poland; 7 Department of Emergency Medical Services, Wroclaw Medical University, Parkowa 34 Str, 51-616 Wroclaw, Poland; 8 Department of Cardiology, 4th Military Hospital, Weigla 5 Str, 50-981 Wroclaw, Poland; 9 Department of Cardiology, 4th Military Hospital, Weigla 5 Str, 50-981 Wroclaw, Poland; 10 Department of Non-Procedural Clinical Sciences, Wroclaw University of Science and Technology, Hoene-Wrońskiego 13 C Str, 58-376 Wroclaw, Poland

**Keywords:** Tipiracil hydrochloride, Ischemia/reperfusion, Thymidine phosphorylase, Metalloproteinases, Drug discovery, Medical research, Neurology, Neuroscience

## Abstract

Thymidine phosphorylase (TP) expression is increased in neurons under ischemia/reperfusion (I/R) conditions. Our aim was to evaluate the effect of tipiracil hydrochloride (TPI), a selective TP inhibitor, on rat brain tissue subjected to I/R. Both common carotid arteries were occluded for 30 min in the ischemic untreated group of rats (C-IR), and ischemic groups treated with tipiracil 25 mg/g (T-IR25) or 50 mg/kg (T-IR50). In the control group (C), the arteries were not ligated. Tipiracil was given during ischemia, and after 8 h of I/R intraperitoneally. After 24 h of I/R, brain tissue was isolated for histology and immunohistochemy of TP expression. Metalloproteinases 2 and 9 (MMP-2 and -9) and tissue inhibitor of metalloproteinases (TIMP-1) were determined in serum at 3 and 24 h of reperfusion. TP expression in brain tissue was the highest in C-IR and T-IR25 compared to the C and T-IR50**.** No changes in serum TP levels were observed. After 24 h, there was a significant decrease in MMP-9 levels in T-IR25 compared to the C-IR and T-IR50. MMP-2 levels also decreased significantly at this time point in all groups compared to group C, which correlated with increased TIMP-1 activity in the T-IR25 and T-IR50. The inhibition of TP activity in the group receiving TPI suggests its protective effect on brain tissue under I/R conditions. The decrease in MMP activities in the treated groups suggests a protective effect of TPI on the development of neuroinflammation caused by local brain tissue ischemia.

## Introduction

Ischemic stroke remains the leading cerebrovascular cause of death and long-term disability worldwide, despite substantial progress in its prevention and treatment^1^. Temporary vascular occlusion in the brain tissue leads to characteristic changes associated with ischemia/reperfusion (I/R) injury. These changes include oxidative stress with the production of reactive oxygen species, disturbances in nitric oxide synthesis and metabolism, the emergence of an inflammatory response with the release of pro-inflammatory cytokines (such as interleukin-1β, interleukin-6, tumor necrosis factor, increased expression of adhesion molecules, activation of microglia, and the migration and adhesion of neutrophils to capillary endothelial cells in the ischemic area of the brain tissue^2,3^. Although timely restoration of blood flow by mechanical or pharmacological means is currently the mainstay of acute stroke therapy, there is an urgent need to identify novel strategies that can effectively mitigate I/R-induced damage and improve patient outcomes.

In this context, modulation of thymidine phosphorylase (TP) activity appears to be an attractive therapeutic target. TP, also known as platelet-derived endothelial cell growth factor or as gliostatin, is an enzyme that catalyzes the reversible phosphorolysis reaction of 2’-deoxythymidine to 2-deoxy-D-ribose −1-phosphate and thymine, and it additionally exhibits deoxyribosyl transferase activity^4,5^. While TP activity has primarily been studied in the context of tumor growth^6–8^, there is evidence that its expression is increased in neurons in brain tissue subjected to I/R injury^9,10^. Our previous LC–MS proteomic analysis of human platelets also revealed elevated TYMP levels that persist for the first three days after ischemic stroke onset^11^. Moreover, both in vivo and in vitro studies have demonstrated the involvement of TP in platelet activation and thrombus formation^12,13^. TP is also a potent inhibitor of glial cells, maintaining homeostasis and protecting neurons in the central nervous system^14^. Together with vascular endothelial growth factor (VEGF), astrocyte-derived TP is thought to be a key factor in the permeability of the blood–brain barrier (BBB)^15,16^. While some reports suggest that TP may promote BBB disruption in conditions such as experimental autoimmune encephalomyelitis and multiple sclerosis^15^, other studies indicate that, unlike VEGF, TP does not increase vascular permeability and that its deficiency may actually enhance cerebral edema formation^17^.

Consequently, selective inhibition of TP could offer protection against I/R-induced brain injury by reducing platelet aggregation and thrombus formation while preserving BBB integrity and glial cell function. Tipiracil hydrochloride (TPI; 5-chloro-6-(2-iminopyrrolidin-1-yl) methyl-2,4 (1H, 3H)-pyrimidinedione hydrochloride) is a potent, competitive TP inhibitor^18^.

This compound is currently used in anticancer chemotherapy in combination with trifluridine. By inhibiting TP, TPI prevents the phosphorylation and degradation of trifluridine, thereby enhancing its anticancer activity^19^. It is plausible that TPI could also protect the structure and function of brain tissue damaged by I/R.

The present study aimed to investigate whether TPI exerts a protective effect on brain tissue subjected to I/R injury through inhibition of TP activity. Specifically, we assessed changes in TP expression in brain tissue by immunohistochemistry and evaluated histopathological alterations. In addition, we measured serum levels of matrix metalloproteinases (MMP-2 and MMP-9) and their tissue inhibitor (TIMP-1), key mediators of the neuroinflammatory cascade that contribute to BBB disruption and neuronal injury in stroke^18,19^. To the best of our knowledge, this is the first study to evaluate the effects of TPI on TP expression and circulating metalloproteinase levels in a rat model of cerebral I/R injury. As a pilot study, the obtained results should be considered preliminary and will serve as a foundation for further experimental work.

## Materials and methods

### Experimental animals

The study was conducted on male Wistar rats aged 10 − 12 weeks. The animals were housed under laboratory conditions, including a 12:12-h light–dark cycle, humidity maintained at 45 − 60%, continuous ventilation, and a temperature range of 21 − 23 °C to ensure the well-being of the animals. Two individuals were housed in each enriched cage.

All procedures used in the study adhered to the ethical standards set by the research committee. The study was reported in accordance with ARRIVE guidelines for animal care and use, as well as methods of data presentation. The experimental protocol received approval from the Local Ethics Committee for Animal Research at the Institute of Immunology and Experimental Therapy of the Polish Academy of Sciences in Wroclaw (No. 031/2020 of May 20, 2020).

### Chemicals

Isoflurane (Isotek, solution 1000 mg/g, 250 ml, Vet-Argo Trading, Lublin, Poland) was used for anesthesia during the isolation and ligation of the common carotid arteries as well as for euthanasia. It was administered by inhalation, at a concentration of 4% for induction and 2% for maintenance.

Butorphanol tartrate (Morphasol, amp. 4 mg/ml, aniMedica GmbH, Frankfurt, Germany) was also used for anesthesia and analgesia during ischemia, after animal recovery during reperfusion, and during euthanasia. It was administered intramuscularly at a dose of 2 mg/kg body weight (b.w.).

Meloxicam (Metacam, sol. 5 mg/ml, 50 ml, Boehringer Ingelheim, Warsaw, Poland) was used for analgesia, administered subcutaneously at a dose of 1 mg/kg b.w. during reperfusion.

TPI (*5-chloro-6-[(2-imino-1-pyrrolidinyl)methyl]−2,4(1H,3H)-pyrimidinedione, monohydrochloride*, C₉H₁₁ClN₄O₂ • HCl, CAS: 183204–72-0, Merck, Kenilworth, NJ, USA) was diluted in 0.9% sodium chloride solution to a final volume of 5 ml/kg (at 5 mg/ml and 10 mg/ml concentrations) and administered intraperitoneally at 10 min of ischemia and 8 h post-reperfusion.

0.9% sodium chloride solution (Polpharma S.A., Starogard Gdański, Poland) was used to restore vascular volume.

### Experimental design

After the 2-week adaptation period, rats were divided into four groups: group C (n = 12) –sham-operated and C-IR (n = 9) – where animals were not treated with TPI, and groups T-IR25 (n = 10) and T-IR50 (n = 9) – where animals received TPI at doses of 25 and 50 mg/kg intraperitoneally, respectively. This compound was administered twice: after 10 min of ischemia and 8 h of reperfusion. Brain tissues were subjected to the I/R procedure in groups C-IR, T-IR25, and T-IR50. Blood samples were taken from the tail vein before the surgical procedure.

Rats were randomly allocated to experimental groups. All surgical procedures were performed by the same experienced team blinded to group assignment. Histopathological and immunohistochemical evaluations were conducted by two independent pathologists blinded to the experimental groups. Biochemical analyses were performed in a blinded manner. The number of animals in the study was limited to the necessary minimum and was determined by the reliability of statistical analysis methods. Assuming an average population value of one of the monitored parameters for Wistar rats at 4 h of reperfusion, aged 10–12 weeks, of 0.73 ± 0.17 μmol/L, and a minimum expected difference of 0.21, with a Type I error probability (α) of 0.05 and a target test power (β) of 0.8, the required sample size (n) is 12. Calculations were performed using STATISTICA Software (version 13.1, from StatSoft, Inc). In our experiment using the bilateral common carotid artery occlusion (BCCAO) model, we achieved a survival rate of 17–25%, resulting in group sizes of 9–10 rats. The observed survival rate falls within the range reported in the literature^20,21^**.**

The TPI doses were selected based on previous in vivo studies in tumor-bearing mice, in which these doses effectively inhibited TP activity without significant toxicity^22^, combined with allometric scaling for rats^23^. PI was administered at two time points: 10 min after the onset of ischemia to achieve peak plasma concentrations during the ischemic phase, and at 8 h of reperfusion to cover the early reperfusion window, when oxidative stress and inflammation peak.

### I/R procedure

Rats were anesthetized with inhaled isoflurane and intramuscularly administered butorphanol (2 mg/kg i.m.). Under general anesthesia, both common carotid arteries were isolated from the median approach in the neck. These vessels were then dissected from the surrounding tissues and isolated from the vagus nerve fibers. Ischemia was induced by placing microclips on common carotid arteries for 30 min (BCCAO). After this period, the microclips were removed, the skin was sutured, and the animals were awakened and observed.

During both ischemia and after 8 h of reperfusion, rats in the T-IR25 and T-IR50 groups received TPI intraperitoneally at doses of 25 or 50 mg/kg, respectively, diluted in 5 ml/kg of normal saline. Blood samples were taken from the tail vein into heparin-coated tubes at 3 and 24 h after the start of reperfusion. After each 0.5 ml blood draw, the vascular volume was replenished with an equal volume of normal saline solution. Twenty-four hours after reperfusion, the animals were euthanized by cervical dislocation at the C6-C7 level under general anesthesia with isoflurane and butorphanol. and brain tissue was isolated, weighed, and fixed in formalin for histopathological and immunohistochemical analysis.

In group C, rats underwent the same anesthesia and surgical procedure as in the ischemic groups, except that the common carotid arteries were not occluded, but only isolated. Blood samples were taken at the same time points as in the ischemic groups. At 24 h after sham surgery, brain tissue was isolated using the same procedure as in the ischemic groups. The same experienced research team blindly performed all surgical procedures.

### Histopathological studies

Various regions of brain tissue obtained from rats subjected to I/R and non-ischemic brains from the control group (sham-operated) without prior selection were fixed in 10% formalin and embedded into paraffin blocks. Using a microtome, 3-μm thick sections were cut from the blocks and mounted on Superfrost Plus slides (Menzel Gläser, Braunschweig, Germany). One section was stained with hematoxylin and eosin according to routine protocols, and the other sections were examined immunohistochemically. Histopathological examination of brain tissue was performed under a light microscope to determine the severity of ischemic necrosis/necrobiosis, apoptosis, neutrophil infiltration, vascular congestion, and architectural destruction.

### Immunohistochemical studies

Staining was performed on a LEICA BOND-MAX (Leica Biosystems, Newcastle upon Tyne, UK) according to the following protocol. First, tissues were deparaffinized (Bond Dewax Solution, Leica Biosystems, Newcastle upon Tyne, UK) and pre-treated with the Bond Epitope Retrieval Solution 2 (Leica Biosystems, Newcastle upon Tyne, UK) for 20 min. Endogenous peroxidase activity was blocked with Peroxide Block using the BOND Polymer Refine Detection System (Leica Biosystems, Newcastle upon Tyne, UK). To measure the level of the studied antigen, the Thymidine Phosphorylase Monoclonal Antibody P.GF.44C (MA5-13,542, Invitrogen, ThermoFisher Scientific, Waltham, MA, US) was applied at a concentration of 1:200 for 15 min at room temperature. The antibody was diluted by Bond Primary antibody Diluent (Leica Biosystems, UK). The samples were then incubated with Post Primary and Polymer using the BOND Polymer Refine Detection System (Leica Biosystems, Newcastle upon Tyne, UK). The 3,3’-diaminobenzidine (DAB chromogen) served as a substrate for the reaction, and all the sections were counterstained with hematoxylin (Leica Biosystems, Newcastle upon Tyne, UK). Control experiments were performed in the absence of the primary antibody. Microscopic photographs were subjected to computer-assisted image analysis using a computer coupled with an Olympus BX53 optical microscope (Olympus, Tokyo, Japan) equipped with a digital ColorView IIIu digital camera (Olympus, Tokyo, Japan). Measurements were performed using cell^A software (Olympus Soft Imaging Solution GmbH, Münster, Germany).

The immunohistochemical reaction was evaluated at 400 × magnification in 10 fields of view. The median number of cells showing a positive reaction (x) was calculated and classified according to the following ranges: (+ + +) intense cytoplasmic reaction in nerve cells and endothelium—more than 15 cells in the field of view show reaction at the highest magnification, (+ +) moderate cytoplasmic reaction in nerve cells and endothelium—up to 15 cells in the field of view show the reaction at the highest magnification, (+) weak cytoplasmic reaction in nerve cells and the endothelium—up to 10 cells in the field of view show the reaction at the highest magnification, (-) no reaction, according to accepted standards^22,23^. Evaluation of immunohistochemical staining in the cytoplasm, nucleus, or cell membrane is inherently subjective and is routinely performed by experienced pathologists.

### Plasma assays

The protein level for TP was determined using ready-to-use MBS053239 ELISA Kits (MyBioSource, Inc., San Diego, CA, USA). The biochemical ELISA technique of that kit is based on TP antibody-TP antigen interactions (immunosorbency) and the HRP colorimetric detection system to detect TP antigen targets in samples.

The Quantikine Rat Total MMP-2 (MMP200) and MMP-9 (RMP900) immunoassays (R&D Systems Europe, Abingdon, UK) were used to measure total rat metalloproteinase-2 (MMP-2) and −9 (MMP-9), respectively. They contain NS0-expressed recombinant mouse/rat pro-MMP-2 and rat MMP-9, respectively.

To determine the levels of natural rat tissue inhibitor of metalloproteinases 1 (TIMP-1) in serum, the Quantikine Rat TIMP-1 Immunoassay (RTM100, R&D Systems Europe, Abington, UK) was used according to the instructions provided with the kit.

### Statistical analysis

Data distribution was assessed using the Kolmogorov–Smirnov test, and homogeneity of variance was evaluated with Levene’s test. When the assumptions of normality and homogeneity were not met, data were logarithmically transformed to meet the requirements of ANOVA. Fisher’s LSD post hoc test was applied only following a significant ANOVA, as it provides greater sensitivity for detecting differences between specific groups, and results are presented as mean ± standard deviation (SD). Non-normally distributed or heteroscedastic data were evaluated using the Kruskal–Wallis H test followed by Dunn’s post hoc test, and presented as medians with 95% confidence intervals. The effects of TPI treatment and reperfusion time on serum parameters were analyzed using repeated-measures ANOVA. The differences were considered significant when p ≤ 0.05. All statistical analyses were conducted using STATISTICA Software (version 13.3, StatSoft, Inc., Kraków, Poland).

**Fig. 1:**
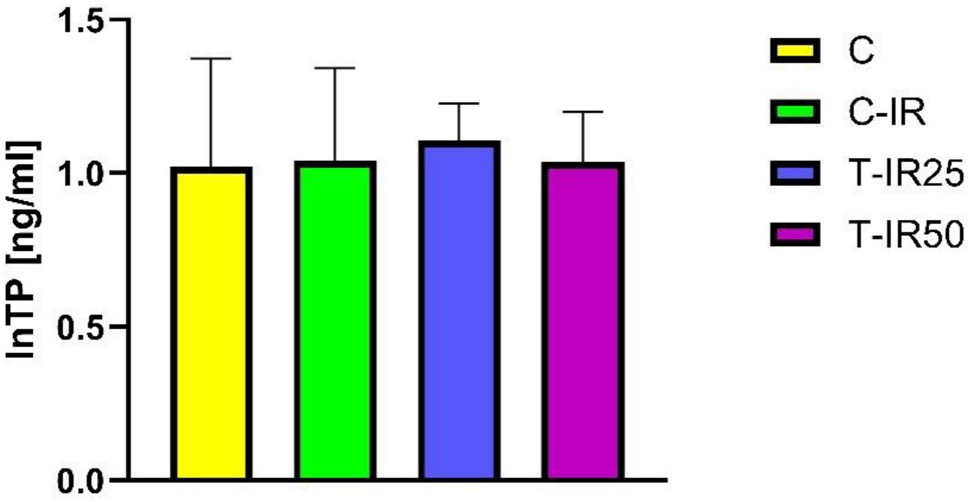
The effect of I/R and TPI treatment on TP protein levels in rat serum was measured by ELISA. Values are expressed after logarithmic transformation as mean ± SD; TP – thymidine phosphorylase, C (n = 12) – control group, C-IR (n = 9) – ischemic and untreated group, T-IR25 (n = 10) – ischemic group treated with TPI at 25 mg/kg dose, and T-IR50 (n = 9) – ischemic group treated with TPI at 50 mg/kg dose. No significant differences were found..

**Fig. 2:**
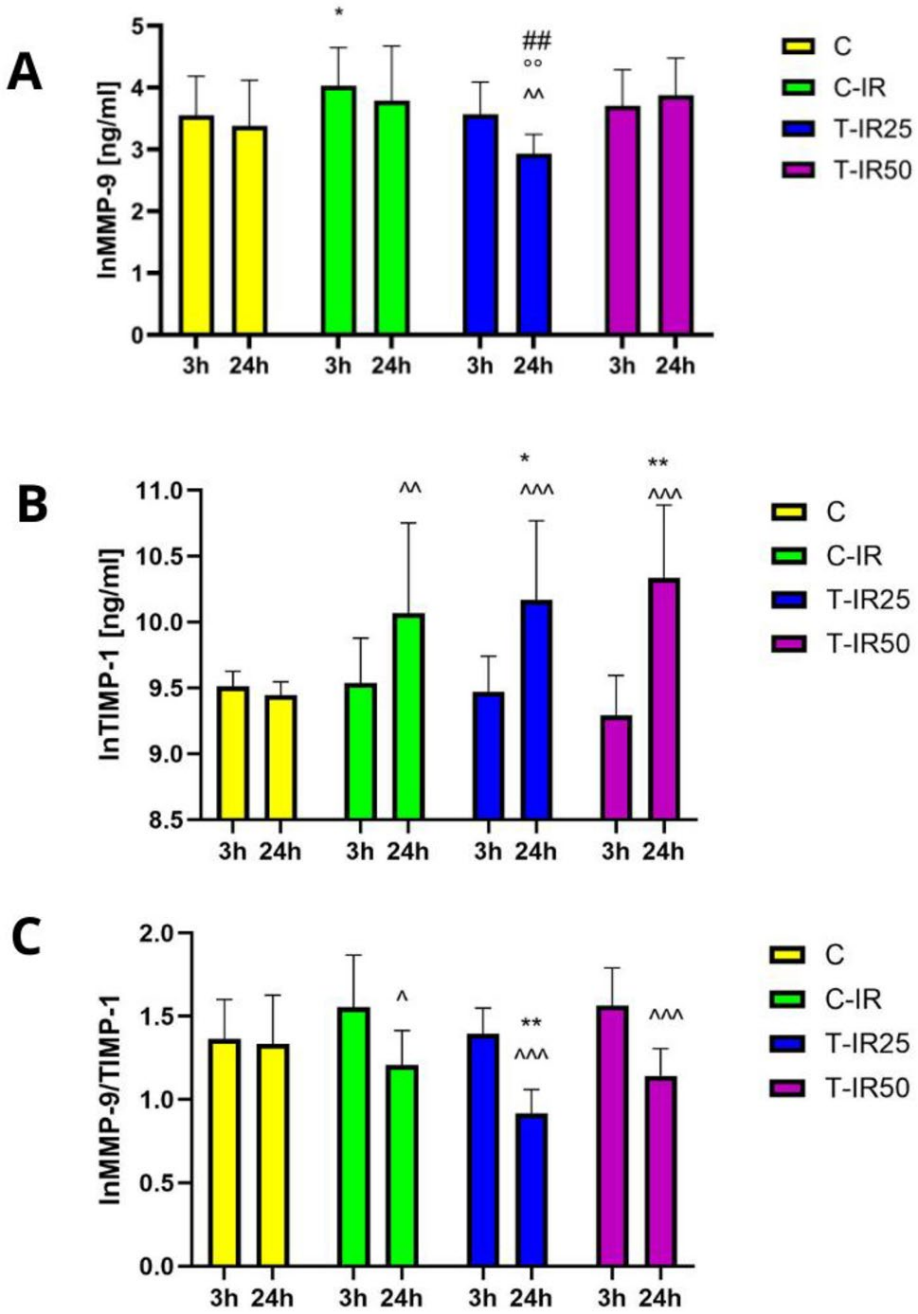
Influence of the I/R and TPI treatment on the levels of MMP-9 (**A**), TIMP-1 (**B**) protein, and MMP-9/TIMP-1 ratio (**C**) in rat serum. Values are presented after logarithmic transformation as mean ± SD; MMP-9 – Metalloproteinase-9, TIMP-1 – Tissue Inhibitor of Metalloproteinases-1, C (n = 12) – control group, C-IR (n = 9) – ischemic and untreated group, T-IR25 (n = 10) – ischemic group treated with TPI at 25 mg/kg dose, and T-IR50 (n = 9) – ischemic group treated with TPI at 50 mg/kg dose. Significant differences: *p < 0.05, **p < 0.01 (vs. C group), ## p < 0.01 (vs. C-IR group), °° p < 0.01 (vs. T-IR50 group), and ^ p < 0.05, ^^ p < 0.01, ^^^ p < 0.001(between the 3-h and 24-h groups).

**Fig. 3:**
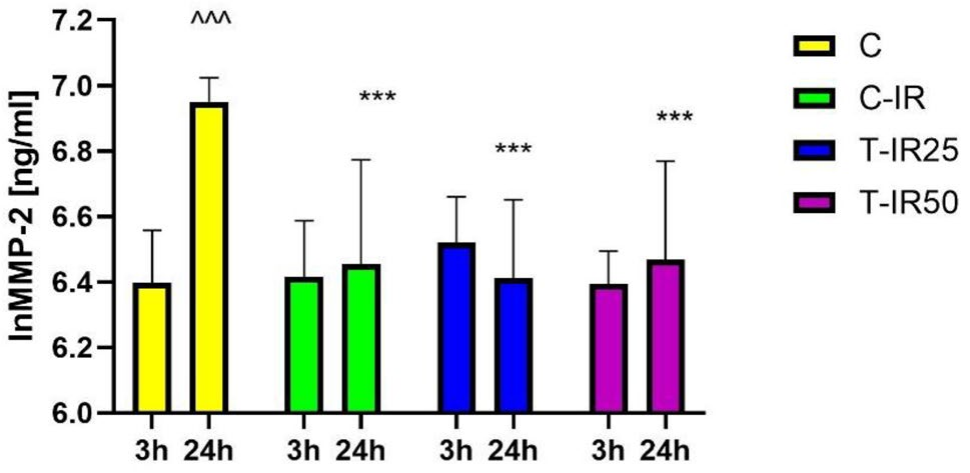
Influence of I/R and the TPI treatment on the MMP-2 protein levels in rat serum. Values are presented after logarithmic transformation as mean ± SD; MMP-2 – Metalloproteinase-2, C (n = 12) – control group, C-IR (n = 9) – ischemic and untreated group, T-IR25 (n = 10) – ischemic group treated with TPI at 25 mg/kg dose, and T-IR50 (n = 9) – ischemic group treated with TPI at 50 mg/kg dose. Significant differences: *** p < 0.001 (vs. C group), and ^^^p < 0.001 (between the 3-h and 24-h groups).

**Fig. 4:**
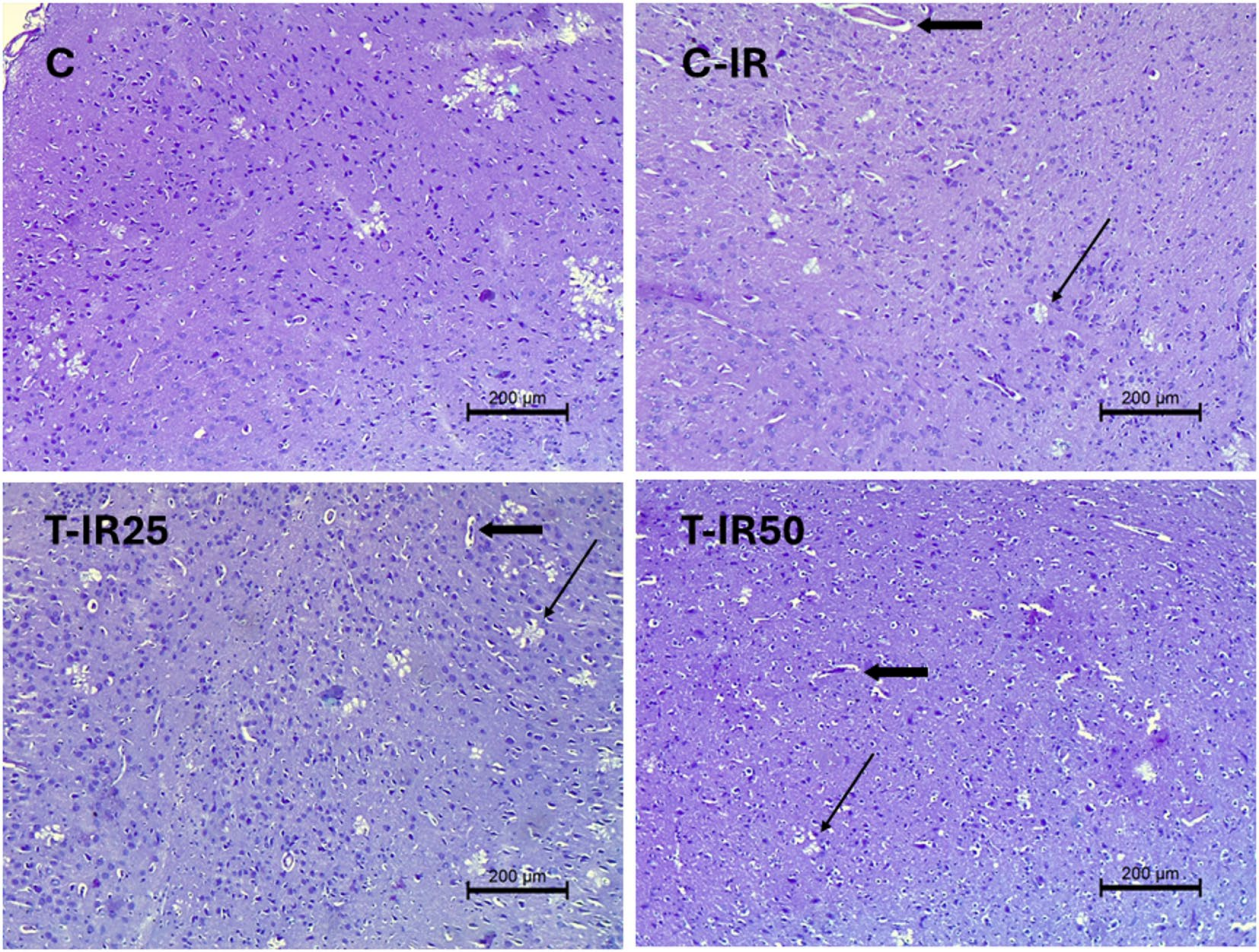
Representative hematoxylin and eosin-stained samples of rat brain tissue (×40) were used to evaluate the morphological changes in all groups: C (n=12) – control group (normal structure), C-IR (n=9) – ischemic and untreated group, T-IR25 (n=10) – ischemic group treated with TPI at 25 mg/kg dose, and T-IR50 (n=9) – ischemic group treated with TPI at 50 mg/kg dose (perivascular edema [wide arrow], vacuolization of individual cells [narrow arrow]).

**Fig. 5:**
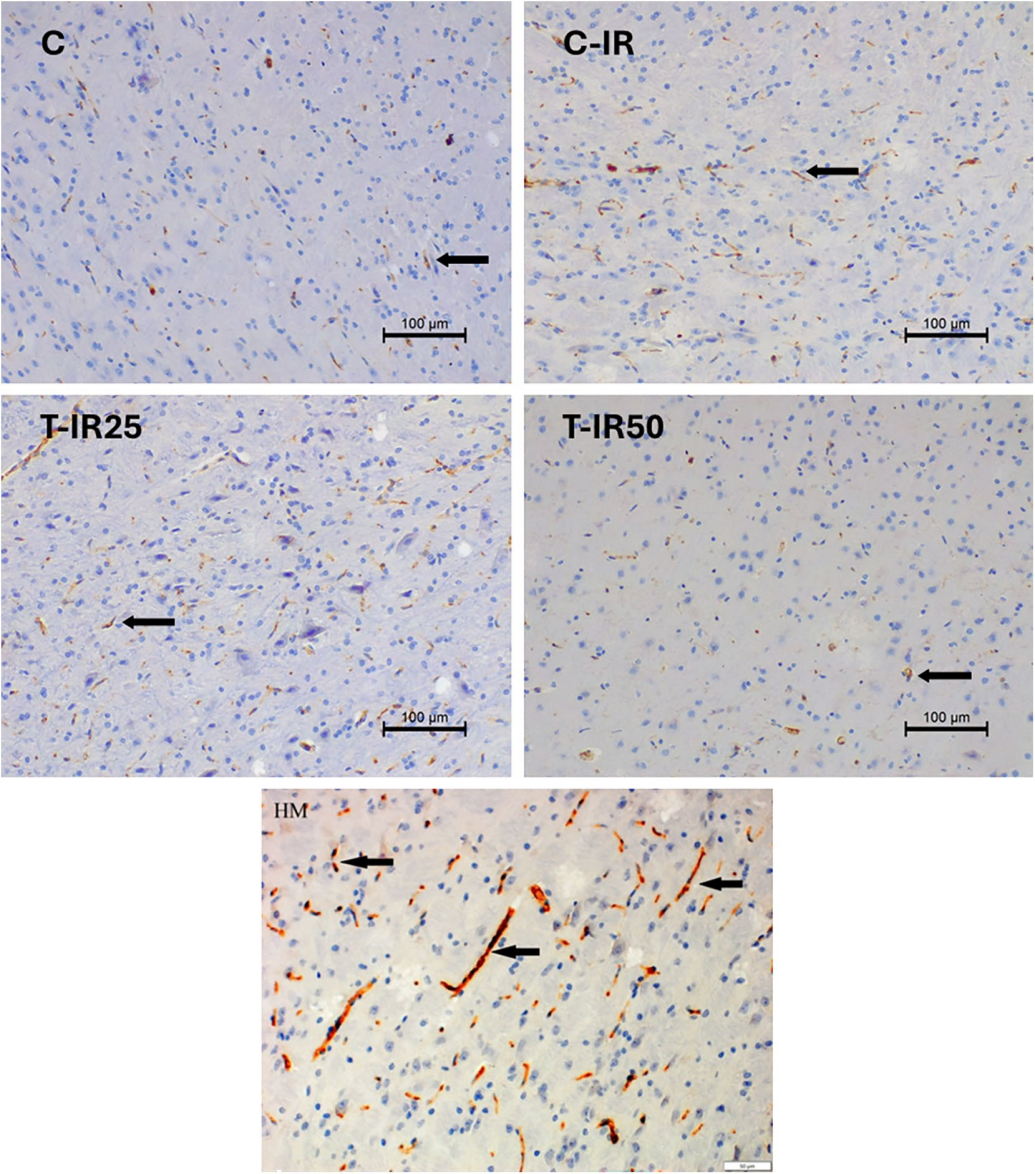
Expression of TP in brain tissue visualized by immunohistochemical reaction (×100). The intensity of the cytoplasmic reaction was assessed in neurons and endothelium (see text). C (n=12) – control group (few small cells were weakly stained [arrow]), C-IR (n=9) – ischemic and untreated group, T-IR25 (n=10) – ischemic group treated with TPI at 25 mg/kg dose, and T-IR50 (n=9) – ischemic group treated with TPI at 50 mg/kg dose (number of positively stained cells was increased, the most in the group (C-IR) and the group (T-IR25[arrow]). A higher magnification (HM) to highlight the immunohistochemical reaction (×200). The arrow indicates the reaction.

**Fig. 6:**
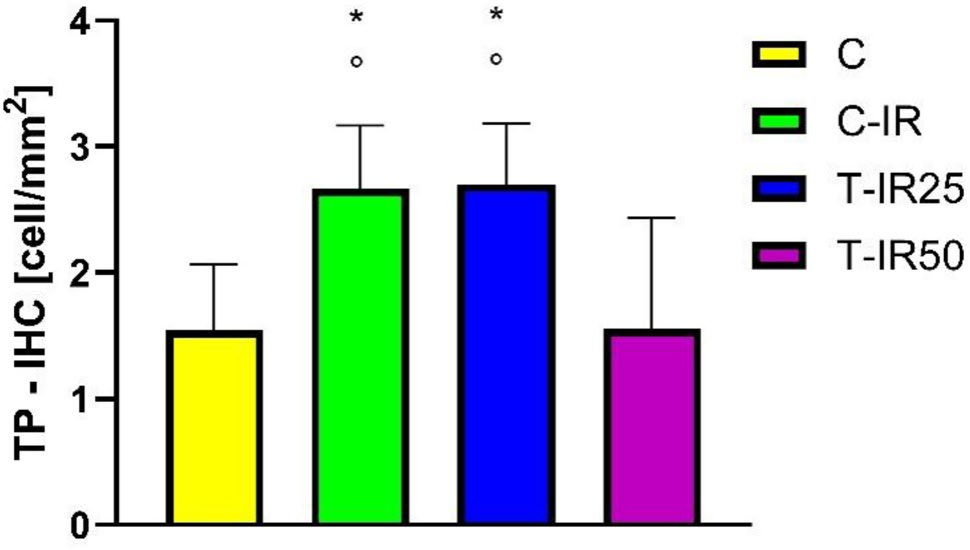
Influence of I/R and the TPI treatment on the expression of TP in rat brain tissue. Values are pre-sented as mean ± SD; TP – thymidine phosphorylase, IHC – immunohistochemistry, C (n = 12) – control group, C-IR (n = 9) – ischemic and untreated group, T-IR25 (n = 10) – ischemic group treated with TPI at 25 mg/kg dose, and T-IR50 (n = 9) – ischemic group treated with TPI at 50 mg/kg dose; specific comparisons: * p < 0.05 (vs. C group), and °p < 0.05 (vs. T-IR50 group).

Figures 1–6 were prepared using the GraphPad Prism version 8.0 (GraphPad Software, San Diego, CA, USA).

## Results

### Blood tests

Twenty-four hours after reperfusion, the serum TP levels in the serum of rats in the C group and those treated with a higher dose of TPI were comparable (p = NS in all comparisons) (Fig. 1).

The highest MMP-9 protein level was observed at 3 h of reperfusion in the untreated I/R group (C-IR: 4.03 ± 0.51 ng/ml), which was significantly higher than in the sham-operated group (C: 3.55 ± 0.63 ng/ml; p = 0.028). At 24 h of reperfusion, MMP-9 levels decreased significantly in the low-dose TPI group (T-IR25: 2.93 ± 0.31 ng/ml) compared with both the untreated I/R group (C-IR: 3.79 ± 0.88 ng/ml) and the high-dose TPI group (T-IR50: 3.88 ± 0.60 ng/ml) (p = 0.0038). It was also significantly lower than the value measured at 3 h in the same T-IR25 group (3.5 ± 0.53 ng/ml; p = 0.004) (Fig. 2A).

At 24 h of reperfusion, a significant increase in TIMP-1 levels was observed in both TPI-treated groups ((T-IR25: 10.17 ± 0.6 pg/ml) and (T-IR50: 10.34 ± 0.55 pg/ml)) compared to the sham group (C: 9.44 ± 0.1 pg/ml) (p = 0.025 and p = 0.001, respectively) and compared to the levels obtained in these groups at 3 h of reperfusion ((T-IR25: 9.47 ± 0.27 pg/ml) and (T-IR50: 9.29 ± 0.3 pg/ml)) (p < 0.001 in both comparisons). Also in the untreated ischemic group (C-IR: 10.07 ± 0.69 after 24 h and 9.54 ± 0.34 after 3 h), a significant increase was observed between those points of time (p = 0.002) (Fig. 2B).

Consequently, the MMP-9/TIMP-1 ratio decreased at 24 h in all I/R groups, with the most pronounced reduction in the low-dose TPI group (T-IR25: 0.91 ± 0.14), which was significantly lower than in the sham group (C: 1.33 ± 0.29; p = 0.002). When the changes in values of this ratio during the reperfusion time were analyzed within groups, significant decreases in the MMP-9/TIMP-1 levels were found between 3 and 24 h of reperfusion in all I/R groups (C-IR: 1.21 ± 0.2 after 24 h and 1.55 ± 0.31 after 3 h, p < 0.05; T-IR25 0.92 ± 0.14 after 24 h and 1.39 ± 0.15 after 3; T-IR50: 1.14 ± 0.16 after 24 h and 1.56 ± 0.23 after 3 h; p < 0.001 in T-IR25 and T-IR50 groups) (Fig. 2C).

The highest concentration of MMP-2 protein was measured 24 h after reperfusion in the control group (C; 6.94 ± 0.07 ng/ml). It was significantly higher in this group compared to all ischemic groups, regardless of drug presence (C-IR: 6.45 ± 0.32 ng/ml, T-IR25: 6.41 ± 0.24 ng/ml, T-IR50: 6.47 ± 0.3 ng/ml; p,0.001 in all comparisons), and compared to the level of this parameter within the control group (C: 6.4 ± 0.16; p < 0.001) observed at 3 h of reperfusion (Fig. 3).

### Histological and immunohistochemical findings

No significant differences in the brain tissue structure were observed in either non-ischemic or ischemic groups after 24 h of reperfusion, regardless of TPI treatment. The brains of the non-ischemic groups showed normal structure. In the ischemic groups, only minor pathological changes were observed. These changes included slight widening of the perivascular spaces, which exhibited features of perivascular edema. Additionally, there was vacuolization of individual cells of the central nervous system, irrespective of the TPI treatment (Fig. 4).

The expression of TP in brain tissue visualized by immunohistochemical reaction, considering the percentage of positive cells and the intensity of the reaction, is summarized in Table. In brains from the sham-operated rats, only a few small cells were weakly stained, but 24 h after reperfusion, the number of positively stained cells was increased, the most in the untreated ischemic group (C-IR) and the group treated with a lower dose of TPI (T-IR25). Examples of the immunoreactivity of the TP are presented in Fig. 5. In brains from the sham-operated rats, only a few small cells were weakly stained, but 24 h after reperfusion, the number of positively stained cells was increased, the most in the untreated ischemic group (C-IR) and the group treated with a lower dose of TPI (T-IR25) Table 1.

**Table 1:** The semi-quantitative evaluation of the TP expression in an immunohistochemical reaction is determined by the percentage of positive cells, which is represented by points of the percentage of positive cells (according to the scale from Remmele et al**.**^24^).

TP expression	Group of rats			
	C	C-IR	T-IR25	T-IR50
Absent (–)	0%	0%	0%	0%
Mild (+)	45.5%	0%	0%	66.7%
Moderate (+ +)	54.5%	40%	30%	11.1%
Intense (+ + +)	0%	60%	70%	22.2%

Cytoplasmic reaction in nerve cells and endothelium: (+ + +) intense – more than 15 cells in the field of view show reaction at the highest magnification, (+ +) weakly intense – up to 15 cells in the field of view shows the reaction at the highest magnification, (+) weak – up to 10 cells in the field of view shows the reaction at the highest magnification, (–) no reaction. C represents the control group, C-IR represents the ischemic and untreated group, T-IR25 denotes the ischemic group treated with TPI at 25 mg/kg, and T-IR50 represents the ischemic group treated with TPI at 50 mg/kg.

The TP expression in the brain tissue of rats was higher in the ischemic and untreated group (C-IR: 2.6 ± 0.5) and the group treated with a lower dose of TPI (T-IR25: 2.7 ± 0.48) than in the sham group (C: 1.55 ± 0.52) (p = 0.023 and p = 0.013, respectively), and the group treated with a higher dose of TPI (T-IR50: 1.56 ± 0.88) (p = 0.049 and p = 0.03, respectively). In contrast to the serum measurements, these differences were statistically significant in brain tissue. The TP expression in the group receiving a higher dose of TPI (T-IR50) was comparable to that in the group not subjected to ischemia (C) (Fig. 6).

## Discussion

Despite many years of in-depth research into the pathophysiology of ischemic stroke and its treatment methods, it remains the leading cause of death and disability in patients worldwide. Therefore, the search for new methods for prevention, diagnosis, and treatment of ischemic stroke appears to be an important focus for further scientific research.

TP plays an important role in various physiological and pathological processes in the human body, including nucleoside degradation and the pyrimidine salvage pathway. In platelets, it is involved in platelet activation and thrombus formation, processes that are critical in the pathogenesis of diseases such as myocardial infarction, stroke, and pulmonary embolism^25^. In addition, the enzyme also stimulates angiogenesis^9^, inhibits apoptosis^26^, and promotes endothelial cell proliferation^27^, as has been studied in solid tumors. Increased TP expression has also been observed in neurons of brain tissue subjected to I/R^9^. Our previous proteomic studies also revealed persistently elevated TYMP levels in human platelets during the first three days after ischemic stroke [A]. Therefore, modulation of this enzyme in both brain tissue and blood appears to significantly affect patient survival after ischemic stroke. This effect could be achieved by protecting neurons in the ischemic area and reducing the systemic propensity for clot formation associated with platelet activation.

Tipiracil hydrochloride (TPI) is a potent and selective competitive inhibitor of TP^15^. Although clinically approved only as a component of anticancer therapy (where it enhances the bioavailability of trifluridine by preventing its degradation^17^), its potential effects in cerebral I/R have not been previously explored. In the present study, TPI was administered as monotherapy to rats intraperitoneally at 25 mg/kg and 50 mg/kg twice during ischemia and 8 h after reperfusion. The results of this experiment indicate a significant effect of TPI on TP and the inflammatory response. Specifically, TPI significantly reduced TP expression in brain tissue at the higher dose of 50 mg/kg, without affecting serum TP levels. It also decreased MMP-9 levels at the lower dose and increased the concentration of the metalloproteinase inhibitor TIMP-1 at both doses, resulting in alterations in the MMP-9/TIMP-1 ratio. In addition, a decrease in MMP-2 levels was observed in the I/R groups 24 h after reperfusion, independent of TPI treatment.

Previous studies using carotid artery thrombosis models in mice and in vitro platelet experiments have shown that TP inhibition prolongs the time to occlusive thrombosis by reducing platelet adhesion and aggregation, without affecting platelet count or general hemostasis^10^. Our findings support this concept, as the higher dose of TPI markedly suppressed TP expression in ischemic brain tissue to levels comparable with sham controls. The minimal changes in serum TP concentration suggest that TPI acts preferentially in ischemic brain tissue, potentially offering a favorable safety profile with limited systemic effects. This discrepancy may also indicate that the serum assay used to quantify TP is insufficiently sensitive or that the changes in brain tissue are too localized to be reflected systemically. However, it is worth noting that the time required for changes in tissue expression may be shorter compared to systemic effects, such as alterations in protein levels. Additionally, tissues subjected to I/R injury may exhibit greater sensitivity, making the effect detectable in brain tissue but not in serum.

Ischemia is known to induce angiogenic factors, including TP, which stimulates the formation of blood vessels. In the present study, transient cerebral ischemia led to increased TP expression in brain tissue. Similar results were obtained by Hayashi et al^9^., who observed an increasing expression of TP in brain neurons during reperfusion. Notably, neither our study nor that of Hayashi et al. revealed increased vascular permeability or cerebral edema on histopathological examination, which aligns with reports indicating that TP, unlike VEGF, does not promote edema formation^17^. The development of brain edema during ischemic stroke therapy with angiogenic factors may become a significant problem. Conversely, inhibition of TP by TPI might theoretically impair its angiogenic or neuroprotective properties, available data do not support harmful effects of TPI in this regard. In tumor models, TPI inhibited tumor growth without suppressing angiogenesis^28^. Therefore, it would be crucial to thoroughly investigate the impact of inhibiting TP growth by TPI on angiogenesis and vascular permeability after transient cerebral ischemia in subsequent reperfusion.

There is increasing evidence that MMPs, including MMP-2 and MMP-9, play a significant role in the pathophysiology of ischemic stroke. They are involved in the early neuroinflammatory cascade by degrading extracellular matrix components, which account for up to 20% of the brain volume^29,30^, increasing blood–brain barrier permeability, and promoting neuronal injury^31,32^. Conversely, during later recovery phases, MMPs, similar to disintegrin and metalloproteinase (ADAMs), play a crucial role in neuronal repair mechanisms^33^ by influencing vascular remodeling, synaptic plasticity, and neural stem cell migration^34^. In our study, we measured metalloproteinase levels at 3 and 24 h after reperfusion to show changes between the early and late stages of reperfusion.

Importantly, the present work is the first demonstration that TPI modulates serum levels of MMP-9 and TIMP-1 in cerebral I/R injury. The observed dose-dependent reduction in MMP-9 and increase in TIMP-1 suggest a novel pleiotropic mechanism of TPI beyond TP inhibition. The highest serum MMP-9 level was observed at 3 h of reperfusion in the untreated ischemic group, whereas MMP-2 levels remained low throughout the reperfusion period. This is consistent with previous studies indicating that MMP-9 increases early after stroke, while MMP-2 elevation predominates in later phases^35^. In this study, Planas et al. showed that the MMP-9 level increased from 4 h to 4 days after ischemia. However, MMP-2 showed only a slight increase at 4 h of reperfusion, whereas a significant increase in expression and activation of this protein was observed in the second phase, approximately 4 days later. The unexpected increase in MMP-2 protein levels on the following day in the control group, in which the cervical vessels were not ligated, is noteworthy. This may indicate the influence of minimal surgical manipulation, including general anesthesia, tissue discontinuity, and vessel isolation in rats, even though ischemia was not induced.

MMPs are regulated at several levels, including by TIMPs, small glycoproteins that form non-covalent complexes with metalloproteinases. TIMP-1 is known to primarily inhibit MMP-9^36,37^, and the MMP-9/TIMP-1 ratio is considered to better reflect MMP-9 activity in vivo^38^. Our results indicate a significant increase in TIMP-1 at 24 h, which was accompanied by a decrease in the MMP-9/TIMP-1 ratio in all ischemic groups. These findings indicate that the neuroprotective potential of TPI may be mediated, at least in part, through upregulation of this endogenous MMP inhibitor. The precise mechanism underlying the stronger effect of the lower TPI dose on MMP-9 remains to be elucidated, but it may involve modulation of oxidative stress and pro-inflammatory cytokines such as TNF-α^40^.

A substantial body of research has been dedicated to the use of synthetic MMP inhibitors. However, these studies have yielded conflicting results due to the occurrence of numerous side effects, which is a salient concern given the critical role of MMPs in the functioning of healthy cells. One such inhibitor is minocycline. In rats with middle cerebral artery occlusion, treatment with minocycline demonstrated a significant decrease in the expression of MMP-2 and MMP-9 in the brain compared to the control group^39^. However, the findings from animal studies and clinical studies with minocycline in ischemic stroke are contradictory^29^. Notably, some studies have demonstrated that prolonged inhibition of MMP in animal models of cerebral ischemia reduces functional recovery and increases brain damage^34,40^. Consequently, therapeutic interventions aimed at inhibiting MMP-9 activity should be considered within the initial 24 h following the onset of an ischemic event.

A notable limitation of the present study was the lack of prominent histopathological changes despite biochemical evidence of I/R injury and TPI effects. This may be explained by the relatively short ischemia duration. Authors of various publications^20,41^ suggested extending the ischemic period to at least one hour. Additionally, it is worth noting that the CCAO model itself may yield highly variable results due to the presence of well-developed posterior communicating arteries in rats. This anatomical feature allows up to 60% of cerebral blood flow to be supplied by the vertebrobasilar system^21,41^. Consequently, the resulting ischemia may have been transient, resembling a clinical transient ischemic attack (TIA) in humans, where no visible changes in the central nervous system (CNS) are observed after 24 h. The most reliable approach may be to modify the experimental model to include occlusion of four arteries—both common carotid and vertebral arteries^42^. However, this procedure is technically more **c**hallenging for the operator, places a greater physiological burden on the animals, and is associated with significantly higher mortality^43^. Another factor worth considering is the type of anesthesia used. Isoflurane has been reported to have neuroprotective effects, potentially through apoptosis inhibition^44,45^. Nonetheless, the absence of discernible changes in histopathological evaluation does not preclude the presence of pathological alterations at the cellular level. The experimental model used in our study successfully demonstrated the protective effects of TPI at the cellular level. As this is a pilot study, further research is necessary to confirm its efficacy and elucidate its underlying mechanisms.

## Conclusions

In summary, this is the first study to evaluate the effect of TPI, a selective TP inhibitor, on TP expression in brain tissue and on serum levels of metalloproteinases (MMP-2 and MMP-9) and TIMP-1 in a rat model of cerebral I/R injury. Given its favorable safety profile, including well-characterized pharmacokinetics, low toxicity, and established clinical approval^17^, TPI warrants further investigation as a potential therapeutic agent in ischemic stroke.

## Data Availability

The raw data supporting the findings of this article are deposited with the authors and will be available upon request.
